# How to Choose the Right Mate

**DOI:** 10.1371/journal.pgen.1003881

**Published:** 2013-10-24

**Authors:** Benoit Arcangioli

**Affiliations:** Institut Pasteur, Dynamic of the Genome Unit, Department of Genomes and Genetics, UMR3525, Paris, France; Brandeis University, United States of America

Genetically programmed recombination plays an important role in differentiation, antigenic variation, and evolution in many systems. Mating-type switching is an example of programmed recombination, and studies of both the budding yeast [1] and fission yeast [2] have provided a wealth of knowledge on how epigenetic and genetic machineries interact with each other to control this process.

Fission yeast has evolved a potent mating-type switching process that rapidly establishes a mixed population, containing roughly the same proportion of P (for plus) and M (for minus) cells, thus allowing sexual reproduction of individuals in a clonal cell population. The sexual region is located on the right arm of chromosome 2 and contains three cassettes—the expressed *mat1* and the two silent *mat2-P* and *mat3-M* loci. Each locus is flanked by the H1 and H2 sequences. The expressed *mat1* locus contains the P genes (Pi and Pc) or the M genes (Mi and Mc) that determine the mating type of the cell [3]. The silent loci located 17 kb from *mat1* are embedded in a 20 kb heterochromatic domain delimited by two boundary elements and enriched in Swi6/HP1 chromodomain protein [4], [5]. The allele present at *mat1* is converted by copying the genetic information from *mat2-P* or *mat3-M* silent donor loci by a non-reciprocal homologous recombination process. Mating-type switching is initiated by a DNA strand-specific imprinting located at *mat1* at the junction between H1 and the specific mating-type allele that is transformed into a polar double strand DNA break (DSB) during DNA replication [6]. The broken DNA does not use the intact sister chromatid, but instead engages recombinational repair by copying the genetic information located between the H1 and H2 sequence of one of the silent donors [7]. This process allows the DNA replication fork to restart, in order to maintain cellular viability and to switch mating-type [8], [9]. Pedigree analysis of switching indicated that this process is very efficient, such that the *mat2-P* donor is favored in M cells and *mat3-M* donor is favored in P cells, with 80% efficiency (reviewed in 2). This intriguing property raises the question of donor preference (i.e., of the directionality of the switching event), which is the topic of a new study by Jakočiūnas et al. [10], published in the current issue of *PLOS Genetics*.

**Figure 1:**
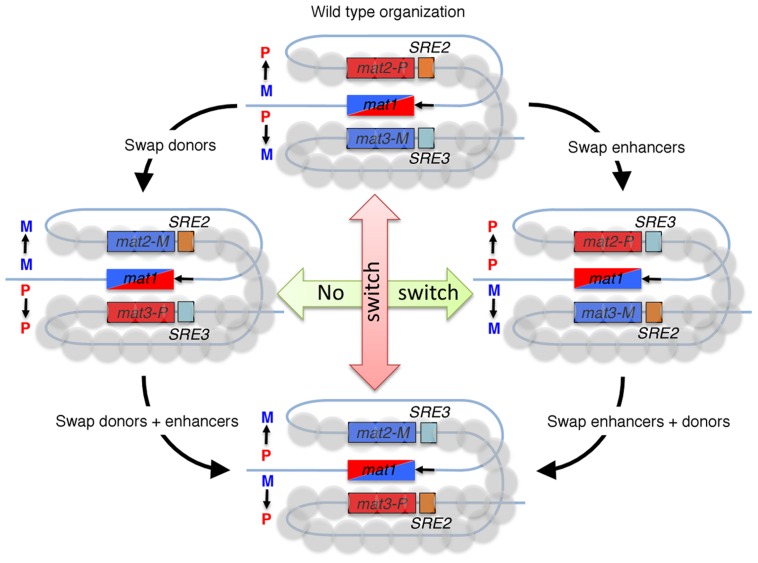
Fission yeast cells switch mating-type in a directional manner, by gene conversions of the *mat1* locus. A recombinogenic DNA end is formed at *mat1* during DNA replication (black arrow) and the broken DNA invades a donor whose genetic information is copied into *mat1*. Wild-type M cells (*mat1-M* allele) use the *mat2-P* donor, while P cells (*mat1-P* allele) use the *mat3-M* donor, as depicted in the top drawing. The recombination enhancers SRE2 and SRE3 are central to these choices. Experiments in which donors and enhancers are swapped, alone or in combination, show that SRE2 and SRE3 are recognized in a cell-type specific manner to promote use of their adjacent donor. The heterochromatic structure of the *mat2-P–mat3-M* region is required for this differential recognition.

In an initial study, Thon and Klar [11] exchanged the alleles present at *mat2* and *mat3* silent donors (*mat2-M, mat3-P*) and found a strong reduction of switching (20%). In the absence of Swi6 or the machinery for histone H3 lysine 9 methylation [12], [13], the switching efficiency becomes random regardless of donor configuration. Collectively, these results demonstrated that the location of the donors, rather than their content, regulates the choice and indicates that the heterochromatic status of the silent donors is critical for the search of the broken *mat1* DNA strand, a process influenced by the mating-type allele present or expressed at *mat1*. Grewal's lab [14] identified a switching recombination enhancer (SRE3) adjacent to *mat3-M* that strongly biases the usage of *mat3* as a donor in P cells and not in M cells. Jakočiūnas et al. have now identified a second enhancer (SRE2) adjacent to *mat2-P*. By swapping the silent cassettes, with or without their cognate enhancers, they showed that these enhancers compete with each other. However, both enhancers are not equivalent with respect to Swi6. Two other switching factors, Swi2 and Swi5 are known to work in the same switching step as Swi6 [15]. Swi2 and Swi5 form a recombination mediator complex. Swi5 is required for general recombination, and Swi2 is specific for mating-type switching, and interacts with itself, Swi5, Swi6, and Rad51, a central protein essential for homologous recombination [16]. In P cells, Swi2/5 is bound to the switching recombination enhancer (SRE3) element, located next to the silent *mat3-M* locus, independently of Swi6. In M cells, Swi2/5 covers the entire silent region and reaches *mat2-P* in a Swi6-dependent fashion [14]. The Grewal lab proposed a spreading model, whereby Swi2/5 complex anchored at SRE3 in P cells will slide onto the Swi6 protein to reach *mat2-P* in M cells. More recently, Grewal's [17] and Klar's [18] laboratories found that the mat1-Mc transcription factor, together with the CENP-B homolog, Abp1 [19], bind the *swi2* gene and regulate its expression. Their results support the notion that the differential distribution of Swi2/5 complex is controlled, at least in part, by mat1-Mc cell type-specific transcription factor. However, it is not clear how the distribution of Swi2/5 over the silent region restricts the usage of *mat2-P* as a donor in M cells. The discovery of the second enhancer thus simplifies the model for directionality (summarized in Figure 1) without the assumption of spreading. Moreover, by using a careful and laborious statistical approach, Jakočiūnas et al. observed a large fluctuation of P/M colonies in *swi2Δ* and *swi5Δ* mutant strains, indicative of inefficient switching, rather than solely a defect in directionality.

It is important to recall that the *Schizosaccharomyces pombe* mutant strain containing a deletion of the *mat2-P* and *mat3-M* region remains fully viable, since the sister chromatid provides the template for *mat1* DSB repair [20]. Thus, the break at *mat1* in the absence of Swi2 (or Swi5) is more likely repaired off the sister chromatid than off the silent donors. As a consequence, the initial allele present at *mat1* of the seeding cells will bias the overall mating-type of the colony, possibly imposing the fluctuation shown by Jakočiūnas et al. Another relevant observation is that the second recombination mediator complex, Rad55/Rad57, also participates in the mating-type switching process [9].

At this stage, it is becoming increasingly clear that several challenging experiments will be necessary to further investigate the mechanism of directionality in fission yeast. State-of-the-art chromosome conformation-capture and classical functional approaches will have to be designed. Moreover, since the heterochromatin association of Swi6/HP1 is regulated during the cell cycle and mating-type switching is triggered only in S-phase, it is important to determine when and how the differential distribution of Swi2/5 (and Rad55/57) complexes is achieved and becomes functional.
